# Tumour site is a risk factor for hepatocellular carcinoma after hepatectomy: a 1:2 propensity score matching analysis

**DOI:** 10.1186/s12893-022-01564-5

**Published:** 2022-03-21

**Authors:** Lian Li, Liangliang Xu, Siqi Zhou, Peng Wang, Ming Zhang, Bo Li

**Affiliations:** Department of Liver Surgery, West China Hospital, Sichuan University, No. 37 Guo Xue Xiang, Chengdu, 610041 Sichuan Province China

**Keywords:** Hepatocellular carcinoma, Anatomic location, Hepatectomy

## Abstract

**Background:**

The effect of the anatomic location of HCC on the prognosis of patients after hepatectomy is currently unclear.

**Methods:**

Patients who underwent hepatectomy were retrospectively enrolled and divided into the right tumour resection group (R group) and the left tumour resection group (L group) according to the tumour anatomic location. To avoid bias, 1:2 propensity score matching (PSM) analysis was used. Based on the survival data, disease-free survival (DFS) and overall survival (OS) were evaluated by the Kaplan–Meier method, and long-term survival analysis was performed. Cox proportional hazards regression was used to analyse the risk factors associated with postoperative prognosis.

**Results:**

A total of 700 patients were enrolled in our study. After 1:2 PSM, 354 and 177 patients were enrolled in the R group and the L group, respectively, with comparable baseline characteristics. Survival analysis showed that patients in the L group had a significantly higher recurrence rate than patients in the R group (P = 0.036), but there was no significant difference in the survival rate (P = 0.99). Long-term survival analysis showed that the survival rate of the L group was lower than that of the R group (P < 0.01). Multivariate analysis showed that tumour location in the left liver was an independent risk factor for tumour recurrence (hazard ratio, 1.263; 95% CI, 1.005–1.587) and long-term survival (hazard ratio, 3.232; 95% CI, 1.284–8.134).

**Conclusion:**

For HCC patients, the recurrence rate and long-term survival rate of left liver tumours were significantly higher than those of right liver tumours, indicating that the anatomical location of the tumour has a significant effect on the survival of HCC patients.

*Trial registration* Chinese Clinical Trial Registry, ChiCTR2100052407. Registered 25 October 2021, http://www.chictr.org.cn/showproj.aspx?proj=135500.

## Background

According to the latest cancer epidemiology survey, liver tumours are the fourth most deadly malignancy in the world, causing 841,080 related deaths in 2018 worldwide [1]. In addition, the death toll from liver tumours continues to rise, and this number is expected to increase to more than one million by 2030 [2]. As the most common malignant tumour, accounting for more than 90% of liver tumours, hepatocellular carcinoma (HCC) has many treatment options [3].

Currently, there are many ways to treat HCC, including hepatectomy, liver transplantation, local ablation and external radiation, transarterial therapies and systemic therapies [4, 5]. Among them, hepatectomy is widely practised in the treatment of HCC and is still an effective radical treatment for select patients [6–8]; however, the 5-year recurrence rate after HCC resection can be more than 70% [9], indicating that the high recurrence rate after surgery greatly limits the treatment of HCC [10]. Many risk factors have been widely reported and recognized for HCC prognosis, such as MVI, tumour size, and tumour number [11–13]. Among the numerous risk factors for recurrence, the relationship between the location of HCC and tumour recurrence has not been fully discussed. Whether there is a difference between the prognosis of left liver cancer and right liver cancer has been rarely discussed. The purpose of this study was to investigate whether the location of HCC affects the prognosis of liver cancer.

## Methods

We retrospectively collected data from 856 patients who underwent hepatectomy at West China Hospital from 2008 to 2015. The diagnostic criteria for HCC referred to the American Association for the Study of Liver Diseases and EASL clinical practice guidelines: Management of HCC [4, 5]. Radical resection of HCC was defined as complete excision of the tumour with clear microscopic margins and no residual tumours demonstrated by computed tomography (CT) scan or angiography at 1 month after surgery [14]. The patients were divided into the left tumour resection group (L group) and the right tumour resection group (R group) according to the site of liver resection. To avoid bias, 1:2 propensity score matching (PSM) analysis between the two cohorts was performed [15]. The inclusion criteria were as follows: 1. patients without other treatment before hepatectomy; 2. hepatectomy limited to only the left or right liver; 3. patients with Child–Pugh stage A or B; and 4. patients with normal cardiopulmonary function and could withstand surgery. The exclusion criteria were as follows: 1. patients with a positive resection margin; 2. patients with recurrence within one month after the operation; 3. Patients who died within three months after surgery; 4. Patients with other malignancies; 5. Patients infected with HCV; and 6. Patients who underwent preoperative radiofrequency ablation or other intraoperative adjuvant treatments. Our study was approved by the West China Hospital of Sichuan University Biomedical Research Ethics Committee.

### Intervention

For patients with a definite preoperative diagnosis, we routinely evaluated whether there were contraindications for surgery. If there were no contraindications, liver tumour resection was performed. Intraoperative ultrasound was routinely used to determine the lesion site, the number of tumours, and the relationship with important intrahepatic structures and to re-evaluate the scope of resection. If new lesions were found, resection or radiofrequency ablation was performed. Patients who underwent only radiofrequency ablation were excluded.

### Follow-up

After the operation, abdominal ultrasound and alpha-fetoprotein (AFP) measurement were regularly performed every 3 months. If suspicious recurrent lesions were detected, contrast-enhanced computed tomography and enhanced magnetic resonance imaging were performed for further evaluation. Overall survival (OS) was defined as the time from the date of surgery to the patient's death or the last follow-up. Disease-free survival (DFS) was defined as the time from the date of surgery to the time of tumour recurrence. Long-term OS in our study were defined as 5-year OS. The end points of follow-up were OS and DFS.

### Statistical analysis

For the clinical characteristics of patients, continuous variables were compared using the unpaired t test or the Mann–Whitney U test, and categorical variables were compared using the chi-squared (X^2^) test or Fisher’s exact test. A 1:2 PSM between the L group and R group was applied to overcome possible selection bias. All of the baseline clinical variables were used in PSM. Patient survival was analysed using the Kaplan–Meier method, and survival curves were compared using the log-rank test. Univariate analyses were carried out using a Cox proportional hazards stepwise model to identify independent factors related to OS and DFS. Stepwise multivariate analysis was performed if the variables were significant (P < 0.1). Analyses were performed by SPSS Statistics version 22.0 for Windows (IBM Corp) and R version 4.1.1 for Windows (32/64 bit).

## Results

### Patient characteristics

**Fig. 1:**
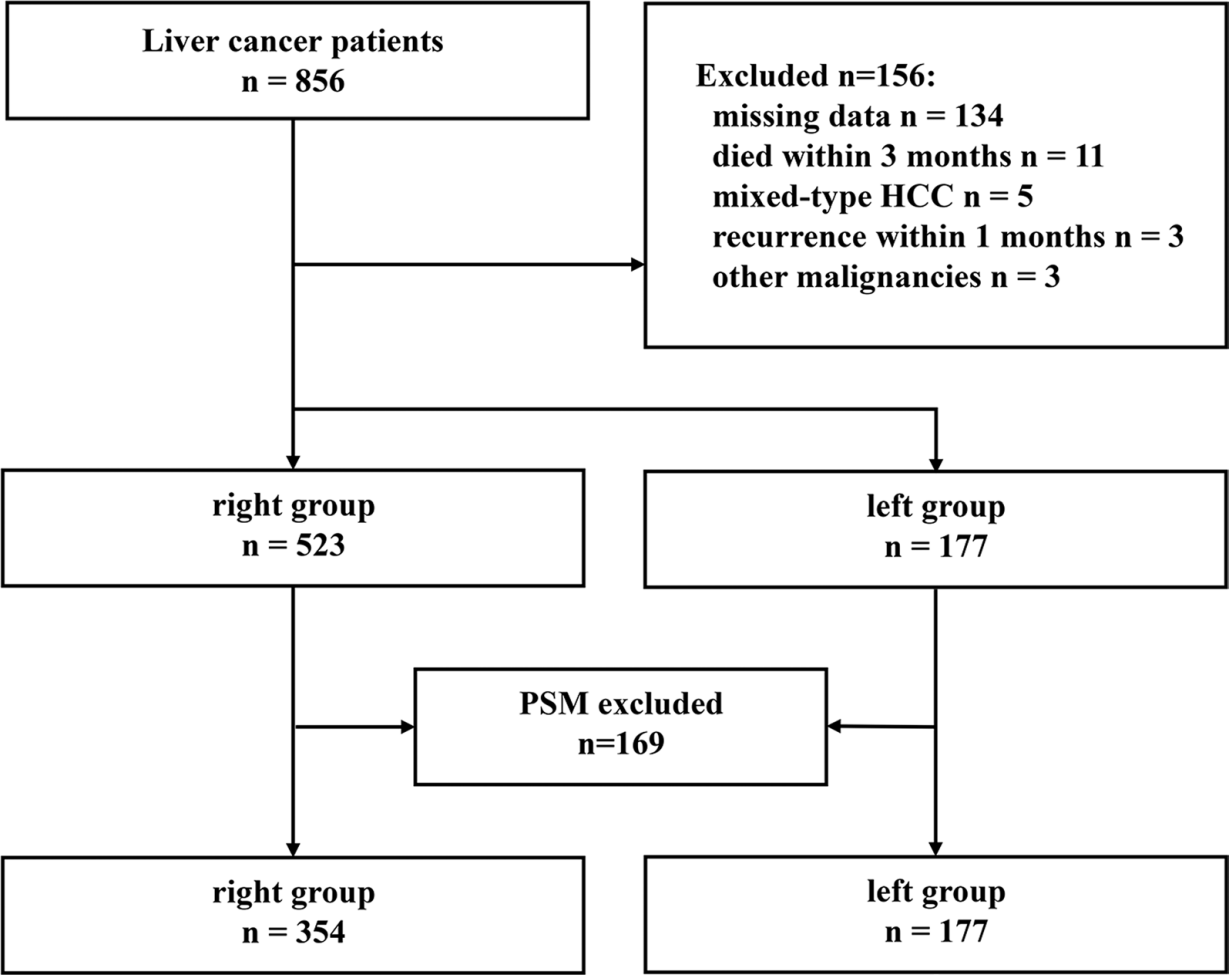
Flow chart of the study participants

**Fig. 2:**
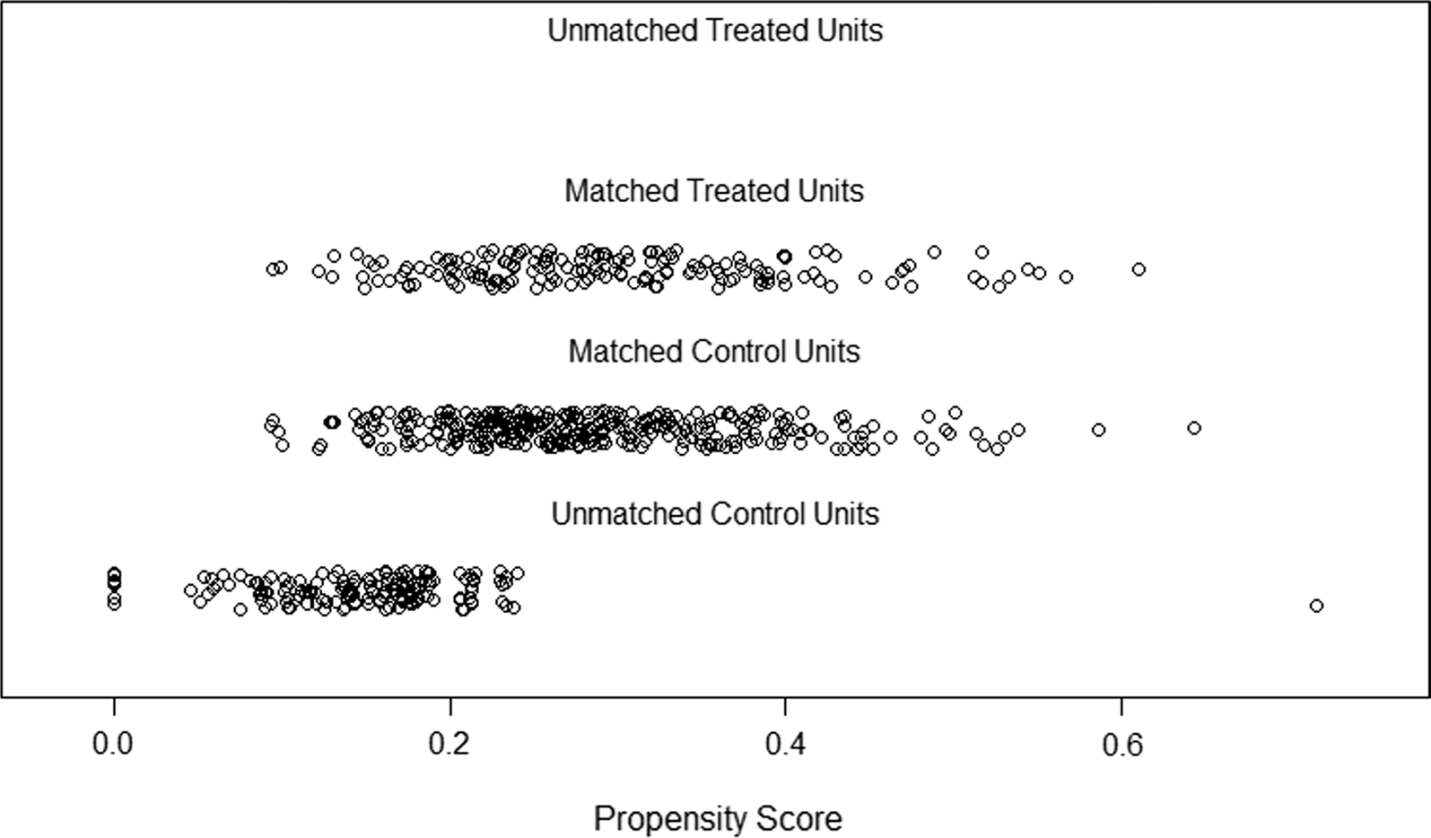
Distribution of propensity scores of L group (Treated Units) and R group (Control Units)

From January 2008 to April 2015, a total of 856 HCC patients who underwent hepatectomy at West China Hospital were retrospectively analysed. Among these patients, 700 patients meeting the inclusion and exclusion criteria were selected for comparison. For the excluded patients, the reasons for exclusion varied, including missing data (n = 134), other malignancies (n = 3), recurrence within 4 weeks (n = 3), death within 3 months (n = 11) and pathological confirmation of mixed-type HCC (n = 5). Finally, a total of 700 patients, consisting of 177 L group patients and 523 R group patients, were enrolled in the analysis. As shown in Table 1, the baseline characteristic data before PSM analysis showed significant differences, including differences in MVI (P = 0.003) and ALT level (P = 0.042). After 1:2 PSM, as shown in Table 2, there were 177 and 354 patients in L group and R group with comparable baseline characteristics (Fig. 1). The detailed scores of matched and unmatched unites in each group are shown in Fig. 2.

**Table 1:** Baseline characteristics before propensity score matching

Variable	R group	L group	P value
	n = 523	n = 177	
Sex (male)	452 (86.4%)	146 (82.5%)	0.246
Poor differentiation	204 (39.0%)	76 (42.9%)	0.291
Lymphatic metastasis	2 (0.4%)	2 (1.1%)	0.573
Cirrhosis	327 (62.5%)	121 (68.4%)	0.191
MVI	156 (29.8%)	75 (42.4%)	0.003
Satellite nodules	77 (14.7%)	24 (13.6%)	0.797
Tumor number (single)	470 (89.9%)	154 (87.0%)	0.220
GVI	41 (7.8%)	18 (10.2%)	0.419
Invading adjacent organs	7 (1.3%)	0 (0.0%)	0.267
Positive HBsAg	463 (88.5%)	151 (85.3%)	0.320
Positive HBeAg	96 (18.4%)	38 (21.5%)	0.424
AFP (< 400 ng/mL)	317 (60.6%)	98 (55.4%)	0.255
Age (year) (IQR)	49.0 (42.0–58.0)	49.0 (41.0–58.0)	0.692
Tumor diameter (cm) (IQR)	5.0 (3.6–8.0)	5.0 (3.5–8.0)	0.772
NEU count (10^9^/L) (IQR)	3.25 (2.46–4.23)	2.99 (2.39–4.09)	0.125
LYM count (10^9^/L) (IQR)	1.45 (1.16–1.80)	1.44 (1.05–1.83)	0.339
PLA count (10^9^/L) (IQR)	129.0 (89.0–184.0)	128.0 (88.5–170.0)	0.373
TBIL level (mmol/L) (IQR)	14.30 (10.70–18.30)	13.40 (10.15–17.75)	0.085
ALT level (U/L) (IQR)	42.00 (28.00–66.00)	39.00 (26.00–57.00)	0.042
AST level (U/L) (IQR)	39.00 (29.00–56.00)	38.00 (29.00–52.00)	0.389
ALB g/dL (IQR)	41.80 (39.10–44.90)	41.60 (39.10–43.95)	0.342
BCLC			
0	235 (44.9%)	86 (48.6%)	0.124
A	209 (40.0%)	55 (31.1%)	
B	37 (7.1%)	19 (10.7%)	
C	42 (8.0%)	17 (9.6%)	

*R group* right tumor resection group, *L group* left tumor resection group, *MVI* microvascular invasion, *GVI* giant vascular invasion, *AFP* alpha fetoprotein, *IQR* interquartile range, *NEU* neutrophil granulocyte, *LYM* lymphocyte, *PLA* platelet, *TBIL* total bilirubin, *ALT* alanine aminotransferase, *AST* aspartate aminotransferase, *ALB* albumin, *BCLC* Barcelona Clinic Liver Cancer Staging

**Table 2:** Baseline characteristics after propensity score matching

Variable	R group	L group	P value
	n = 354	n = 177	
Sex (male)	290 (81.9%)	146 (82.5%)	0.968
Poor differentiation	152 (42.9%)	76 (42.9%)	0.882
Lymphatic metastasis	2 (0.6%)	2 (1.1%)	0.859
Cirrhosis	248 (70.1%)	121 (68.4%)	0.764
MVI	133 (37.6%)	75 (42.4%)	0.330
Satellite nodules	42 (11.9%)	24 (13.6%)	0.676
Tumor number (single)	319 (90.1%)	154 (87.0%)	0.392
GVI	34 (9.6%)	18 (10.2%)	0.959
Invading adjacent organs	0 (0.0%)	0 (0.0%)	1.000
Positive HBsAg	305 (86.2%)	151 (85.3%)	0.895
Positive HBeAg	72 (20.3%)	38 (21.5%)	0.850
AFP (< 400 ng/mL)	205 (57.9%)	98 (55.4%)	0.642
Age (year) (IQR)	49.0 (41.0–59.0)	49 (41.0–58.0)	0.510
Tumor diameter (cm) (IQR)	5.0 (3.5–8.0)	5.0 (3.5–8.0)	0.743
NEU count (10^9^/L) (IQR)	3.12 (2.42–4.13)	2.99 (2.39–4.09)	0.684
LYM count (10^9^/L) (IQR)	1.41 (1.11–1.77)	1.44 (1.05–1.83)	0.983
PLA count (10^9^/L) (IQR)	122.0 (84.0–173.0)	128.0 (88.5–170.0)	0.617
TBIL level (mmol/L) (IQR)	14.00 (10.60–17.83)	13.40 (10.15–17.75)	0.348
ALT level (U/L) (IQR)	40.00 (26.75–57.25)	39.00 (26.00–57.00)	0.618
AST level (U/L) (IQR)	38.00 (29.00–54.00)	38.00 (29.00–52.00)	0.695
ALB g/dL	41.70 (39.00–44.90)	41.60 (39.10–41.60)	0.562
BCLC			
0	175 (49.4%)	86 (48.6%)	0.780
A	118 (33.3%)	55 (31.1%)	
B	29 (8.2%)	19 (10.7%)	
C	32 (9.0%)	17 (9.6%)	

*R group* right tumor resection group, *L group* left tumor resection group, *MVI* microvascular invasion, *GVI* giant vascular invasion, *AFP* alpha fetoprotein, *IQR* interquartile range, *NEU* neutrophil granulocyte, *LYM* lymphocyte, *PLA* platelet, *TBIL* total bilirubin, *ALT* alanine aminotransferase, *AST* aspartate aminotransferase, *ALB* albumin, *BCLC* Barcelona Clinic Liver Cancer Staging

### Association of tumour location and prognosis

**Fig. 3:**
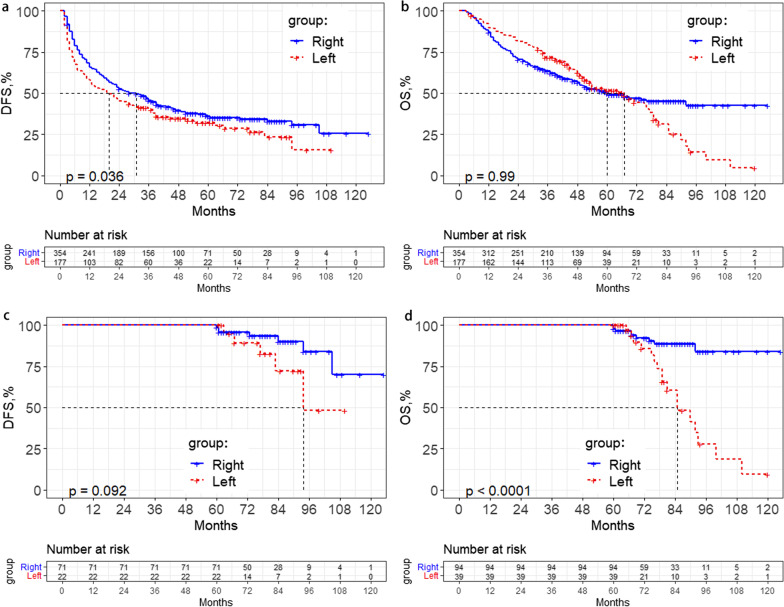
Kaplan–Meier analysis of disease-free survival (DFS) and overall survival (OS) for hepatocellular carcinoma patients with different anatomy locations: **a** DFS for the L group and R group after 1:2 PSM. **b** OS for the L group and R group after 1:2 PSM. **c** Long-term of DFS for the L group and R group after 1:2 PSM. **d** Long-term of OS for the L group and R group after 1:2 PSM

According to the tumour location of the patients before the operation, two groups were investigated: the L group and the R group. After the PSM analysis, for L group patients, the 1-, 3- and 5-year recurrence rates after surgery were 43.4%, 59.5% and 67.9%, while in the R group, the recurrence rates were 34.3%, 54.3% and 63.9%, respectively (P = 0.036) (Fig. 3a). However, this trend was not found for OS, and there was no significant difference between the two groups of patients. For L group patients, the 1-, 3- and 5-year mortality rates after surgery were 10.2%, 28.1% and 48.2%, while those of the R group were 13.0%, 36.4% and 50.2%, respectively (P = 0.990) (Fig. 3b). Multivariate analysis demonstrated that tumour location (hazard ratio, 1.263; 95% CI, 1.005–1.587), lymphatic metastasis (hazard ratio, 6.229; 95% CI, 2.228–17.412), MVI (hazard ratio, 1.711; 95% CI, 1.330–2.202), satellite nodules (hazard ratio, 1.449; 95% CI, 1.018–2.063), HBeAg (hazard ratio, 1.639; 95% CI, 1.255–2.139), and AFP levels (hazard ratio, 1.421; 95% CI, 1.131–1.786) were independent risk factors for DFS (Table 3). For OS, we failed to find that tumour location was an independent risk factor after multivariate analysis.

**Table 3:** Uni- and multivariate analyses of disease-free survival (DFS) and overall survival (OS)

Variable	Univariate		Multivariate	
	HR (95% CI)	P value	HR (95% CI)	P value
DFS				
Location, left vs right	1.271 (1.018–1.588)	0.034	1.263 (1.005–1.587)	0.046
Differentiation, well vs poor	0.712 (0.575–0.882)	0.002		
Lymphatic metastasis, yes vs no	6.729 (2.487–18.210)	< 0.001	6.229 (2.228–17.412)	< 0.001
MVI, yes vs no	2.390 (1.930–2.960)	< 0.001	1.711 (1.330–2.202)	< 0.001
Satellite nodule, yes vs no	2.504 (1.875–3.345)	< 0.001	1.449 (1.018–2.063)	0.040
GVI, yes vs no	3.155 (2.299–4.329)	< 0.001		
BCLC, A vs 0	1.691 (1.325–2.159)	< 0.001		
BCLC, B vs 0	1.979 (1.371–2.856)	< 0.001		
BCLC, C vs 0	2.897 (1.325–5.806)	< 0.001		
HbeAg, positive vs negative	1.481 (1.155–1.899)	0.002	1.639 (1.255–2.139)	< 0.001
AFP, > 400 vs < = 400 ng/mL	1.762 (1.425–2.180)	< 0.001	1.421 (1.131–1.786)	0.003
Age, y (continuous)	0.984 (0.975–0.993)	< 0.001		
Tumor diameter, cm (continuous)	1.092 (1.066–1.119)	< 0.001		
NEU, 10^9^/L (continuous)	1.078 (1.000–1.162)	0.049		
Platelet, 10^9^/L (continuous)	1.003 (1.001–1.005)	< 0.001		
AST, U/L (continuous)	1.004 (1.002–1.006)	< 0.001		
Long-term OS				
Location, left vs right	4.793 (2.132–10.780)	< 0.001	3.232 (1.284–8.134)	0.013
MVI, yes vs no	4.117 (1.897–8.935)	< 0.001	3.161 (1.284–7.786)	0.012
Satellite nodule, yes vs no	3.017 (1.029–8.845)	0.044		
GVI, yes vs no	4.215 (1.566–11.340)	0.004		
BCLC, C vs 0	4.831 (1.696–13.765)	0.003		
AFP, > 400 vs ≤ 400 ng/mL	2.230 (1.016–4.891)	0.046		

*HR* hazard ratio, *CI* confidence interval, *MVI* microvascular invasion, *GVI* giant vascular invasion, *BCLC* Barcelona Clinic Liver Cancer Staging, *AFP* alpha fetoprotein, *AST* aspartate aminotransferase

### Long-term survival analysis

We found potential differences after five years by observing the shape of the survival curve. Therefore, we wondered if different sites had an effect on long-term outcomes and analysed the survival outcome after five years. We did not find a significant difference in long-term DFS. For L group patients, the 5-, 8- and 10-year recurrence rates after surgery were 67.9%, 84.5% and 84.5%, while for R group patients, the recurrence rates were 63.9%, 69.3% and 74.4%, respectively (P = 0.092) (Fig. 3c). However, there were differences in long-term OS; for L group patients, the 5-, 8- and 10-year mortality rates after surgery were 48.2%, 85.7% and 95.2%, while for R group patients, the OS rates were 50.2%, 57.4% and 57.4% (P < 0.01) (Fig. 3d). Multivariate analysis demonstrated that tumour location (hazard ratio, 3.232; 95% CI, 1.284–8.134) and MVI (hazard ratio, 3.161; 95% CI, 1.284–7.786) were independent risk factors for long-term OS (Table 3).

## Discussion

Although many risk factors have been widely reported to influence survival, few previous studies have reported the effect of the location of primary HCC lesions. Previously, surgeons’ understanding of liver anatomy was mainly for the innovation of the surgical method of liver resection. However, with the gradual deepening of understanding, the influence of anatomy on the prognosis of HCC should also be considered. In this study, we enrolled a large group of patients and analysed the relationship between the location of the primary HCC lesion and the survival of patients. We found that the postoperative recurrence rate and long-term mortality rate of the L group were significantly higher than those of the R group.

At present, late recurrence of HCC is generally considered 2 years after surgery [16, 17], while 5 years is usually regarded as the time point for long-term survival [18, 19]. The risk factors of early and late prognosis of HCC after hepatectomy are different and these issues have been discussed by many studies. Imamura et al. conducted research shows that factors related with elevated carcinogenesis, like higher grade of hepatitis activity, multiple tumours, and gross tumour classification, contributed to late phase prognosis [20]. Wu et al. found high viral loads and hepatic inflammatory activity were associated with late recurrence [21]. Zhang et al. revealed compared with women, men had greater late recurrence rate and rate of cancer-specific mortality [22]. From the perspective of liver anatomy, there are different structural systems of left and right hepatic venous outflow. First, most of the left hepatic vein and the middle hepatic vein converge before returning to the inferior vena cava, while the right hepatic vein exists independently [23, 24]. Fang, C. et al. conducted 3D reconstruction of hepatic veins in 200 patients and found a common trunk for the left hepatic and middle veins [25]. These main channels of venous outflow after hepatectomy may be affected to varying degrees among tumour locations, thus causing different degrees of liver congestion. Second, in addition to the different outflows of the main hepatic veins, there were significant differences in the distribution of short hepatic veins between the left and right liver. Mehran R. et al. divided the main short hepatic veins of the liver into four categories, most of which drained the right liver but not the left liver [26]. Furthermore, Sakaguchi et al. specifically analysed intrahepatic venovenous shunts of the right liver by hepatic venography [27], indicating that the right liver usually has a larger number and diameter of short hepatic veins.

The difference in the distribution of the main hepatic vein and the short hepatic vein can cause the difference in the left and right hepatic vein return. At present, whether there is a clear relationship between hepatic congestion and the prognosis of HCC after hepatectomy has not been reported in large numbers of patients, but liver congestion is widely regarded as a risk factor for the recurrence of liver tumours after liver transplantation and the survival of patients [28–30]. Although there is no direct evidence to prove it at present, it has been found in clinical observation that liver congestion after left liver surgery is more serious than that after right liver surgery.

This study has some limitations. First, objectively evaluating liver congestion at present is difficult, and the direct relationship between congestion and tumour prognosis needs to be confirmed by follow-up studies. Second, due to the retrospective nature of the data, many missing clinical data points could not be remedied, and important gaps remain in our knowledge of this process. The results need to be confirmed by prospective studies.

## Conclusion

Tumour anatomic location has a significant impact on the recurrence and long-term survival of HCC patients after hepatectomy, while it does not seem to affect the short-term survival of patients.

## Data Availability

The datasets used and analysed during the current study are available from the corresponding author on reasonable request.

## Abbreviations

R group: Right tumour resection group
L group: Left tumour resection group
PSM: Propensity score matching
DFS: Disease-free survival
OS: Overall survival
CI: Confidence interval
HCC: Hepatocellular carcinoma
MVI: Microvascular invasion
HCV: Hepatitis C virus
AFP: Alpha fetoprotein
GVI: Giant vascular invasion
IQR: Interquartile range
NEU: Neutrophil granulocyte
LYM: Lymphocyte
PLA: Platelet
TBIL: Total bilirubin
ALT: Alanine aminotransferase
AST: Aspartate aminotransferase
ALB: Albumin
BCLC: Barcelona clinic liver cancer staging
